# Exogenous expression of Drp1 plays neuroprotective roles in the Alzheimer's disease in the Aβ42 transgenic *Drosophila* model

**DOI:** 10.1371/journal.pone.0176183

**Published:** 2017-05-22

**Authors:** Fengshou Lv, Xiaopeng Yang, Chuanju Cui, Chunhe Su

**Affiliations:** 1 Department of Pathology, Henan Medical College, Zhengzhou, China; 2 Department of Neurology, The Second Affiliated Hospital of Zhengzhou University, Zhengzhou, China; 3 Department of Neurology, Zhengzhou First People's Hospital, Zhengzhou, China; Texas Technical University Health Sciences Center, UNITED STATES

## Abstract

**Background:**

Alzheimer's disease (AD) is one of the most common neurodegenerative disorders. Recent studies have shown that mitochondrial dysfunction is a causative factor of AD. Drp1 (Dynamin-related protein 1), a regulator of mitochondrial fission, shows neuroprotective effects on Parkinson’s disease. In this study, we investigate the effect and mechanism of Drp1 on Aβ42 transgenic *Drosophila*.

**Methods:**

Elav-gal4/UAS>Aβ42 transgenic *Drosophila* model was constructed using Elav-gal4 promoter. The effects of Drp1 on the lifespan, motor ability and neuronal degeneration of the transgenic *Drosophila* were explored by over-expressing Drp1 in the Aβ42 transgenic *Drosophila*. ATP levels in the brain tissues of Aβ42 transgenic *Drosophila* were detected using high performance liquid chromatography (HPLC).

**Results:**

Exogenous expression of Drp1 promoted crawling ability, reduced the levels of ATP in *Drosophila* brain and suppressed the neuronal degeneration.

**Conclusion:**

The protective effect of Drp1 on the Aβ42 transgenic *Drosophila* was achieved by protecting the mitochondrial function, suggesting that Drp1 may be a potential therapeutic strategies for AD.

## Introduction

Alzheimer’s disease (AD), a degenerative disease of the central nervous system, is the most common type of neurodegenerative disorders [1–4]. AD is characterized by memory deficits and cognitive impairments. More than 36 million people older than 65 years were diagnosed as AD worldwide [1]. Amyloid β (Aβ) plays key roles in AD pathogenesis via regulating neuronal cell death [5, 6]. It was reported that the continuous production, degradation and the aggregation of amyloid beta-protein (Aβ) lead to AD [7]. Aggregation of beta amyloid (Aβ42) and tangles of neuronal fibers are the main pathological features [8,9]. However, the mechanisms driving the disease have not been completely understood.

Mitochondria are dynamic organelles with frequent fusion and fission [10]. A number of studies suggest mitochondrial dysfunction is a key causative factor of AD. Qi et al. and Xie et al. reported that inhibition of mitochondrial fission could protect the neuronal cells against neurotoxicity [11,12]. Therefore, exploring the regulators of mitochondrial dynamics may provide a novel therapeutic target for AD.

Dynamin-related protein 1(Drp1), which belongs to the dynamin GTPase family, is a key regulator of mitochondrial fission [13,14]. Recent evidence suggested Drp1 played important roles in the neuroprotective effects [15,16]. Barsoum et al. and Gomez et al. reported that the hyperactivity of Drp1 induced by dopaminergic neurotoxins could promote neuronal cell death [17,18]. Moreover, in Phillip’s report, blocking the function of Drp1 *in vivo* either through genetic or small-molecule approach is neuroprotective in a mouse model with damage in the nigrostriatal pathway [19]. However, whether Drp1 provides neuroprotection in AD still remains unknown.

In this study, to explore whether Drp1 has a neuroprotective effect on AD, Drp1 was overexpressed in Aβ42 transgenic *Drosophila* nervous system using Elav-GAL4/UAS stains, and investigated whether the protective effects associated with the function of mitochondrial. Our results showed Drp1 may act as a potential therapeutic strategy for AD.

## Materials and methods

### Materials

458 strains of genotype P{w[+mW.hs] = GawB}Elav[C155], abbreviated as (Elav-GAL4 UAS-Drp1-DN), UAS-Drp1-DN DKVc-16 strains and P{UAS-APP.Aβ42.B} strains were purchased from Bloomington *Drosophila* Stock Center in USA. Aβ42 *Drosophila* primase and standard ATP solution were purchased from Tian Di Yang Biotechnology Company and Italy SANT S.R.L Company. BCA protein assay kit, hematoxylin and eosin (HE) staining kit were purchased from Beyotime Biotechnology Research Institute.

### Chromatographic conditions

The mobile phase was prepared according to the appendix of Chinese pharmacopeia. Water: methanol (0.05 mol/L) was 92.5:7.5. 10.88 g KH_2_PO_4_ and 0.8 g NaOH were dissolved in 400 ml and 200 ml ultrapure water respectively. 121.6 ml NaOH solution, 400 ml KH_2_PO_4_ solution and 60 ml methanol solution were diluted with ultrapure water to 800 ml as phase solution. The phase solution was filtered using 0.45 μm filter and degassed in an ultrasonic bath. Flow rate: 1 mL/min, chromatographic column: Pak C184um PNova, detection wavelength: 259 nm.

### Synthesis of nucleotide fragments

According to the cDNA sequence of Aβ42 in GenBank, The primers of Aβ42 gene and Aβ42-CpG fragment were artificially synthesized by Tian Di Yang Biotechnology Company. The sequences of the forward and reversed primers for Aβ42 gene and CpG were 5’-GCAGAATTCCGACATGACTCAG-3’ and 5’-GCCCACCATGAGTCCAATGA-3’ respectively.

### Construction of the Aβ42 transgenic *Drosophila* model

To construct the UAS-Aβ42arc/TM3 transgenic *Drosophila* with W, Elav-GAL4/FM genetic background, the W1118 virgin females and UAS-Aβ42Arc transgenic *Drosophila* were mated with Elav-GAL4 male respectively. Elav-W1118 and Elav-Aβ42 *Drosophila* were collected whthin 24 hours and killed after 25 days. Under the microscope, the *Drosophila* heads were cut by anatomical needle, and the brain tissues were extracted. Then, the expression of Aβ42 was determined by fluorescence PCR technique.

### The life length test of *Drosophila*

The life length test of *Drosophila* was taken at 25°C. When entering eclosion period, the adult flies were killed. 200 newly emerged males were collected within 24 hours and fed with the fresh medium. The number of survival was recorded every 2 days. Each strain was divided into 5 groups and each group of 20 *Drosophila*. To determine whether the life length of *Drosophila* was shortened, the survival rate curve was done according to the average number of survival flies.

### Crawling ability test

When entering eclosion period, 200 newly emerged males were collected within 24 hours and fed with the fresh medium. *Drosophila* creeping tubes were transparent plastic pipes with 30 cm in height, 2 cm in diameter, and 0.1 cm in thickness. The test was taken on 15^th^ and 25^th^ day. Each strain was divided into 5 groups and each group of 20 *Drosophila*. When the test was made, gently shake the tube body so that the *Drosophila* would drop into the bottom of the tubes. Due to the characteristics of negative chemotaxis, the fruit flies will leave the bottom of the tube to crawl. Record the numbers of *Drosophila* at different positions under 5 cm, between 5 and 10 cm and higher than 15 cm. Each group was tested 5 times, and the test interval was 5 minutes. The average number and standard deviation were calculated.

### Detection of Aβ42 in brain

100 newly emerged males were collected within 24 hours and fed with fresh medium. The heads of 50 males drawn randomly were cut by dissecting needle after 25 days. The brain tissues were extracted under a microscope, then fixed in 10% buffered formalin and processed for paraffin sectioning. The sections were routinely dewaxed, hydrated, and stained with hematoxylin and eosin. After staining, the tissue sections were observed with the objective magnifications of 20X40.

### Determination of the content of ATP in the brain of *Drosophila*

The brain tissues were extracted from 50 males drawn randomly on 25th day and ground in a mortar with liquid nitrogen and 200 μl of 0.4 mol/L HClO_4_. Then, the grinding liquid was centrifuged at 3500 rpm for 15 min. KOH was added slowly to the supernatant to adjust the pH to neutral (supernatant: = 200:415 solution KOH ratio). The mixture was then centrifuged at 3500 rpm for 15 min. The supernatant was decanted and stored at -80°C. The level of ATP was determined by HPLC1100. 0.5, 1.0, 2.0, 4.0 and 6.0 ml of 0.1 mg/ml control ATPn and 1 ml internal standard solution were diluted into 10 ml, and then 10 μl of the diluent was injected into the chromatograph. The vertical coordinate indicated the ratio of the ATP to the internal standard peak and the horizontal coordinate indicated the ATP concentration. The regression equation is X = -0.31580+17.09515*Y, r = 0.9995. The results showed good linearity when the concentration of ATP between 5 and 55 mg/L.

### Statistical analysis

All results were expressed by mean ± standard deviation (X ± S). The paired t-test, one-way ANOVA, q test, and correlation analysis were used to compare the values from the experimental group and the controls using SPSS 17 statistical software. The difference was considered as significant when the p-value was less than 0.05.

## Results

### The characters of Elav-GAL4 system Aβ42 transgenic *Drosophila*

**Fig 1:**
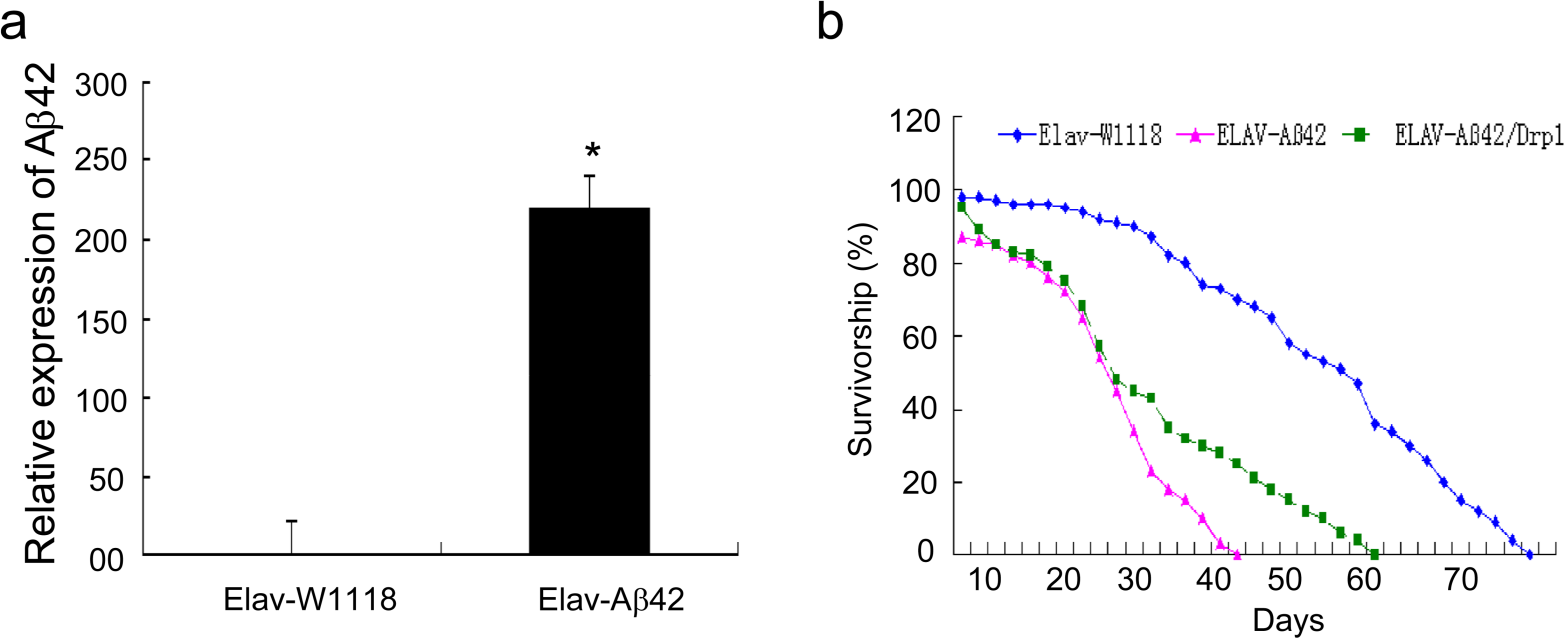
Elav-GAL4 system Aβ42 transgenic *Drosophila*. (a) The control group was wild-type Elav-W1118. The relative expression level of Aβ42 was increased about 200 times in Elav-Aβ42 *Drosophila* detecting by the fluorescent PCR. (b) The average lifetime of Elav-W1118 (72.6 days), Elav-Aβ42 transgenic (41.4 days) and Elav-Aβ42/Drp1 (60.5 days) were recorded as shown by the survival curves. After over-expressing Drp1 in Elav-Aβ42 transgenic *Drosophila*, it was found that the average life of Elav-Aβ42 / Drp1 was longer than that of Elav-Aβ42. The asterisks indicated the p value was lower than 0.05 when compared compared Elav-Aβ42 group to wild-type Elav-W1118 group.

To determine the transgenic efficiency, the relative expression of Aβ42 was detected by fluorescent PCR. The value of the wild-type Elav-W1118 was 1, while that of Elav-Aβ42 was 200 (Fig 1A). According to the survival curve, the average life length of the wild-type Elav-W1118 and Elav-Aβ42 transgenic group were 72.6 days and 41.4 days, respectively. Over-expressing Drp1 extended the average life length of Elav-Aβ42 *Drosophila* from 41.4 days to 60.5 days (Fig 1B).

### Crawling ability test of Elav-GAL4 system Aβ42 transgenic *Drosophila*

**Fig 2:**
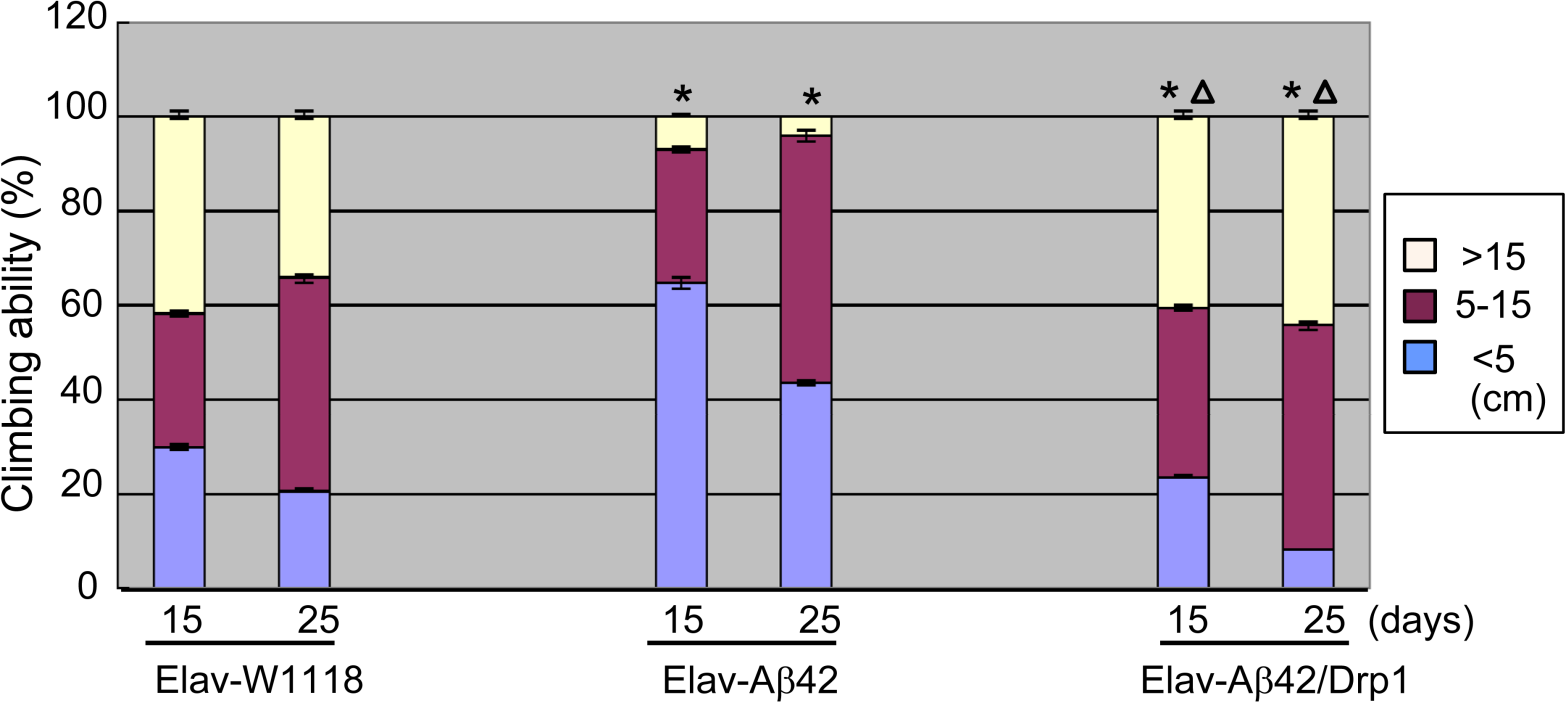
Crawling ability test. The wild-type group: on the 15th day, the average number of Elav-W1118 *Drosophila* whose crawling height within 0–5 cm was 7.68, and the number above 15 cm was 10.45; on the 25th day, the average number within 0-5cm was 5.27, and the number above 15 cm was 8.67. Elav-Aβ42 *Drosophila*: on the 15th day, the average number whose crawling height within 0–5 cm was 15.58, and the number above 15 cm was 1.86; on the 25th day, the average number within 0–5 cm was 11.01, and the number above 15 cm was 1.56. After Drp1 was over-expressed in Elav-Aβ42 transgenic *Drosophila*, on the 15th day, the average number within 0–5 cm was 11.98, and the number above 15 cm was 8.98; on the 25th day, the average number within 0-5cm was 10.68, and the number above 15 cm was 6.87. It was found that after over-expressing Drp1 in Elav-Aβ42 transgenic *Drosophila*, the average number of crawling *Drosophila* within 0–5 cm was decreased and that above 15 cm was increased in the first 15 and 25 days. The asterisks indicated the p value was lower than 0.05 when compared Elav-Aβ42 group or Elav-Aβ42/Drp1 group to wild-type Elav-W1118 group. The triangle indicated that the p value was lower than 0.05 compared between Elav-Aβ42 group and Elav-Aβ42/Drp1 group.

On the 15^th^ day, the average numbers of *Drosophila* whose climbing heights within 5 cm of the wild-type Elav-W1118, model group Elav-Aβ42 and Elav- Aβ42/Drp1 group were 7.68, 15.58, 11.98 respectively. And on the 25^th^ day, the numbers were 5.27, 11.01, and 10.68. On the 15^th^ day, the average numbers of *drosophila* whose climbing heights above 15 cm for the wild-type Elav-W1118, model group Elav-Aβ42 and Elav-Aβ42/Drp1 group were 10.45, 1.86, 1.56. And on the 25^th^ day, the numbers became 5.27, 1.56 and 6.87. Thus, overexpression of Drp1 reduced the average numbers of *Drosophila* whose climbing heights within 5 cm and increased that whose climbing heights above 15 cm (Fig 2).

### Detection of the neuronal degenerative level of Aβ42 transgenic *Drosophila*

**Fig 3:**
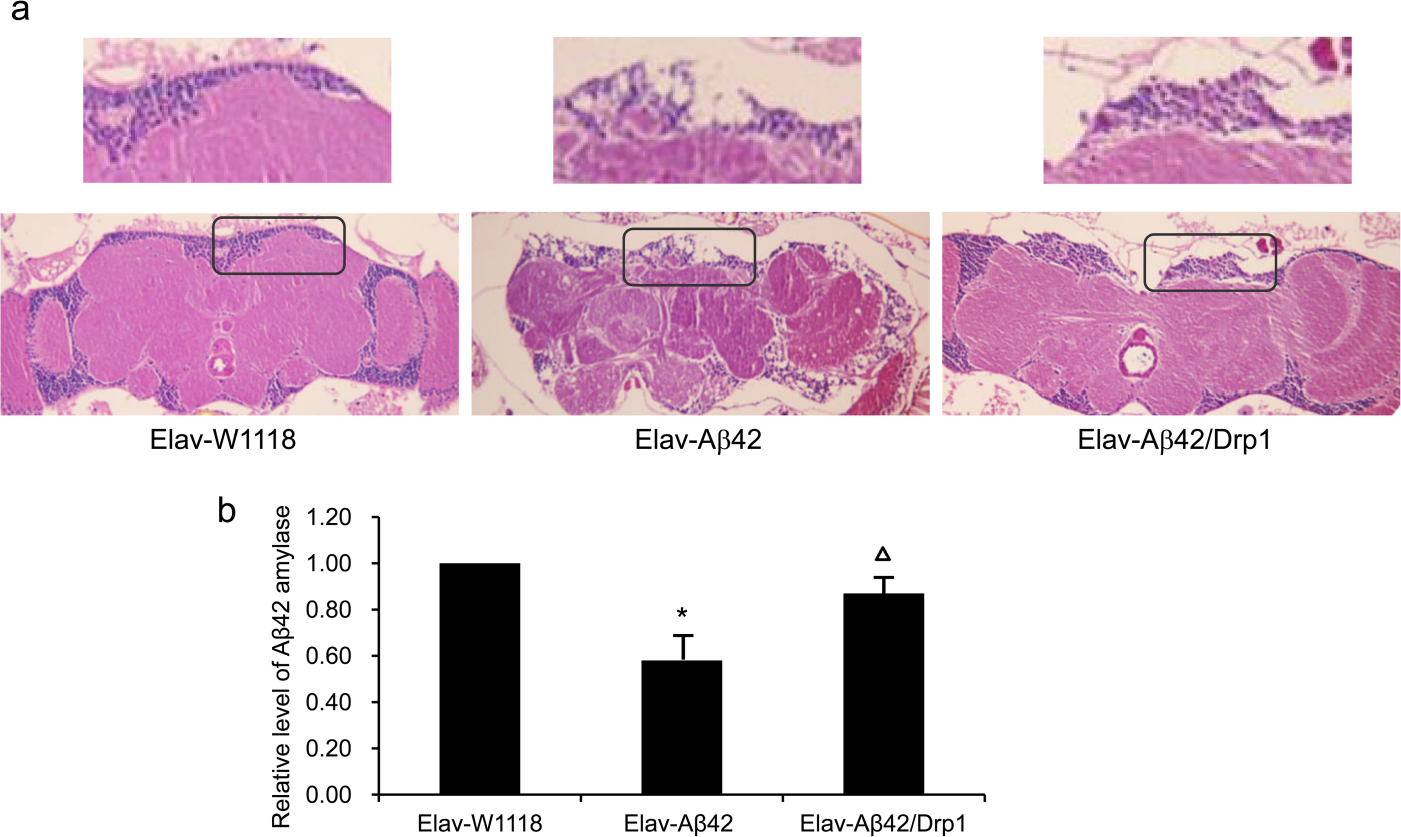
Detection of the neuronal degeneration level. (a) Aβ42 amyloid fibrils detection and routine HE staining of *Drosophila* brain sections were carried out. The figures were observed and captured under the Olympus microscope (20X, 40X), it was found that overexpressing Drp1 in Elav-Aβ42 transgenic *Drosophila* suppressed the neuronal degeneration. (b) The quantitative result for the relative level of Aβ42 amyloid fibrils. The asterisks indicated the p value was lower than 0.05 when compared Elav-Aβ42 group to wild-type Elav-W1118 group. The triangle indicated that the p value was lower than 0.05 compared between Elav-Aβ42 group and Elav-Aβ42/Drp1 group.

Aβ42 amyloid fibrils in *Drosophila* brain tissues were detected by routine HE staining and observed with the Olympus microscope (objective: 20X, 40X). As shown in Fig 3, the exogenous expression of Drp1 in Elav-Aβ42 inhibited the neuronal degeneration.

### The relative levels of ATP in Elav-GAL4 system Aβ42 transgenic *Drosophila*

**Fig 4:**
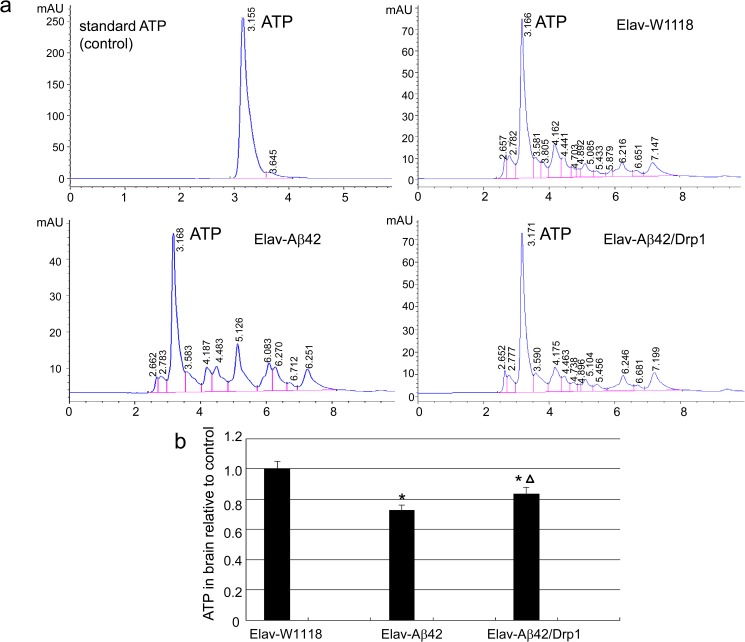
Relative level of ATP of the transgenic Elav-GAL4 system. (a) In the first 25 days, ATP level in *Drosophila* head was measured, ATP peak area of the wild-type *Drosophila* was 114.69 mAU*s; after Drp1 overexpressed in Elav-Aβ42 *Drosophila*, the peak area (163.62 mAU*s) was significantly higher than that of Elav-Aβ42 *Drosophila* (88.61 mAU *s). (b) Similarly, the relative average level of ATP for Elav-Aβ42/Drp1 Drosophila (0.84) was significantly higher than that of *Drosophila* Elav-Aβ42 (0.73). The asterisks indicated the p value was lower than 0.05 when compared Elav-Aβ42 group or Elav-Aβ42/Drp1 group to wild-type Elav-W1118 group. The triangle indicated that the p value was lower than 0.05 compared between Elav-Aβ42 group and Elav-Aβ42/Drp1 group.

The ATP levels in the head of *Drosophilas* were detected on the 25th day. The peak areas of the wild-type group Elav-W1118, model group Elav-Aβ42 and Elav-Aβ42/Drp1 group were 114.69 mAU*s, 88.61 mAU*s and 163.62 mAU*s respectively (Fig 4A). Similarly, the relative level of ATP level of Elav-Aβ42/Drp1 was 0.84, which was significantly increased compared to that of Elav-Aβ42 group (0.73) (Fig 4B).

## Discussion

In this study, we investigated whether Drp1 contributes to the pathogenesis of AD. AD is the most common neurodegenerative disorder. Recently studies suggested that the continuous production, degradation and the aggregation of amyloid beta-protein (Aβ) lead to AD. Aggregation of beta amyloid (Aβ42) and tangles of neuronal fibers are the main pathological features. In this study, we constructed an Aβ42 transgenic *Drosophila* model to explore the mechanisms driving the disease.

Although the pathogeny of AD remains unclear, studies have shown that mitochondrial dysfunction may be one of the causative factors [20,21]. Mitochondria are dynamic organelles. The excessive fusion and fission of mitochondria can lead to the change of size and shape of it. In the cell and mouse models of AD, mitochondrial fragmentation has been reported to contribute to the pathogenesis of AD [9,22].

Mitochondrial fission is controlled by the dynamin-related protein 1 (Drp1), which is a member of the dynamin family of large GTPase. Drp1 was reported to be involved in several important structural features of mitochondria, including shape, size, distribution, remodeling and the maintenance of mitochondria in mammalian cells [23]. Phosphorylation is the most common post-translational modification of Drp1, which regulates its activity. The functional roles of phosphorylation of serine 616 (S616) and serine 637 (S637) in Drp1 have been widely reported. Recently studies suggested important roles of Drp1 in the neuroprotective effects. In AD model mice, the phosphorylation level of Drp1 (Ser616) is significantly up-regulated in the brains cells [24,25]. Barsoum et al. and Gomez et al. also reported hyperactivity of Drp1 inducing by the dopaminergic neurotoxins could promote neuronal cell death [17,18]. However, whether Drp1 contributes to AD pathogenesis remain still unclear.

It was reported that Drp1 cooperates with Aβ to regulate neuronal damage in the AD patients. Manczak et al. found that Drp1 interacts with Aβ monomers and oligomers in AD patients and these abnormal interactions are increased with the disease progression [26]. Dah et al. reported Aβ could also activate Drp1 through sustained phosphorylation of Akt [8]. Thus, it is reasonable to suppose that Drp1-Aβ42 interaction is implicated in the regulatory mechanism of Drp1 in Aβ42 transgenic *Drosophila*.

In this study, we investigated the roles of Drp1 in regulating the pathogenesis of AD by using Aβ42 transgenic *Drosophila*. With Elav-GAL4/UAS promoter, Aβ42 protein was expressed in the *Drosophila* brain neurons. With exogenous expression of Drp1, we found the average life expectancy of Elav-Aβ42 extended to 60.5 days from 41.4 days. We also found that exogenous expression of Drp1 could significantly promote crawling ability and reduced the level of ATP in brain tissues. Moreover, neuronal degeneration was suppressed after Drp1 overexpression. These results suggested that exogenous expression of Drp1 had a neuroprotective effect on Aβ42 transgenic *Drosophila* models.

## Conclusions

Taken together, our study showed that exogenous expression of Drp1 could promote crawling ability, reduced the level of ATP in *Drosophila* brain and suppressed neuronal degeneration, suggesting that Drp1 may be a potential therapeutic strategy for AD disease. We think our study was helpful to explore the pathogenesis of the central nervous system degenerative disease like AD.
